# Validation of the attitudes towards people living with HIV/AIDS scale in nursing students

**DOI:** 10.1186/s12912-023-01414-6

**Published:** 2023-07-27

**Authors:** María Gázquez-López, Inmaculada García-García, Alberto González-García, Adelina Martín-Salvador, María Ángeles Pérez-Morente, Encarnación Martínez-García, María Adelaida Álvarez-Serrano

**Affiliations:** 1 Department of Nursing, Faculty of Health Sciences, University of Granada, Ceuta, Spain; 2 Department of Nursing, Faculty of Health Sciences, University of Granada, Avenida de la Ilustración n. º 16, Granada, Granada 18016 Spain; 3 Department of Nursing, Faculty of Health Sciences, University of Jaén, Jaén, Spain; 4 Guadix High Resolution Hospital, Andalusian Health Service, Granada, Spain

**Keywords:** HIV/AIDS, Attitude of Health Personnel, Nursing students, Psychometrics.

## Abstract

**Background:**

One of the environments where people living with HIV/AIDS should feel safer is in the health care setting; however, scientific evidence has identified discriminatory behaviour on the part of health care professionals towards these people. The reduction or abolition of discriminatory practices requires, first of all, to know the attitudes of nursing students towards AIDS with tools appropriate to the socio-cultural context of the disease. The objectives of this study are to update the AIDS Attitudes Scale for Nursing Students (EASE) by adapting it to the sociocultural landscape and to analyse the reliability and structural validity of the new scale.

**Methods:**

The results of the questionnaires answered by 213 undergraduate nursing students from the Faculty of Health Sciences of Ceuta (University of Granada) were analysed. Reliability (test-retest, n = 33) and validity (n = 180) tests were carried out.

**Results:**

An exploratory and confirmatory factor analysis indicated that a four-factor model was the most parsimonious solution. Items were examined for their underlying relationships and labelled: professional practice, social integration, partner and family, and benevolent stigma. The new scale yielded a McDonald’s Omega coefficient (ω) of 0.893. Convergent validity was established for average variance extracted per factor greater than 0.5 and divergent validity when the variance retained by each factor is greater than the variance shared between them (average variance extracted per factor > ϕ2).

**Conclusions:**

The new scale is a psychometrically sound instrument for assessing attitudes towards people living with HIV/AIDS in nursing students.

**Supplementary Information:**

The online version contains supplementary material available at 10.1186/s12912-023-01414-6.

## Background

Human immunodeficiency virus (HIV) infection has become a chronic disease thanks to antiretroviral treatment (ART). It is estimated that of 38.4 million people living with HIV/AIDS (PLWHA), about 28.7 million had access to ART, a figure that has tripled since 2010 [1]. However, despite the fact that more than 40 years have passed since the first cases were reported [2–5], this infection remains a global public health problem [6–8]. In 2021, an estimated 1.5 million people were newly infected, and an estimated 650,000 people died from AIDS-related illnesses [9].

Despite this pharmacological control of the infection and the time that has elapsed since the first cases were detected, false beliefs and lack of information in the general population generate negative and discriminatory attitudes towards PLWHA and the infection itself, with great repercussions for the people who suffer from it [10–13]. Thus, it has been shown that these stigmatising attitudes and behaviours can impede and decrease the number of diagnosed individuals who are adequately treated and who achieve viral suppression [14]. In addition, patients report that stigma is the main barrier to seeking care, which leads to lack of access and minimal support, and can have a negative impact on treatment adherence and use of health services [15].

Discriminatory behaviour against PLWHA has been documented to come primarily from HIV-negative individuals with close ties to PLWHA [16, 17]. For PLWHA, the most painful experience is the rejection they experience from family and friends [7, 11]. This social amalgam of negative attitudes, stigmatisation and discriminatory behaviour generates fear in PLWHA and they often hide their HIV status as a self-protection mechanism for themselves and their environment [18]. In some cases, concealment results in social isolation, leading to deficits in social support networks and even ostracism [11, 19, 20].

Presumably, one of the safest environments in which a PLWHA should feel safe is in the health care setting [21, 22]. It is in this professional group that competencies such as assertive communication, empathy, ethical management of health care, encouraging active participation of the patient or user and promoting self-management of their own health are assumed [23, 24]. Paradoxically, different studies show that the attitudes of health professionals towards PLWHA tend to be negative as a result of lack of knowledge and fear of HIV/AIDS, which directly affects the quality of care for these patients [22, 25].

Scientific evidence has identified discriminatory behaviours by health professionals towards PLWHA such as asking sarcastic questions, labelling them negatively, spreading their HIV status among colleagues and family members, being upset about having to care for them, to the point of refusing care, taking unnecessary precautions such as wearing double gloves, masks, or burning sheets, advising them not to have sex, marry or start a family, and even holding them responsible for their actions in contracting the infection because of its association with assumed immoral behaviour [6, 7, 17, 25, 26]. In the case of nursing students, their appraisal of PLWHA is also often negative [11, 12, 27].

Several instruments have been developed to measure nursing students’ attitudes towards this context [28–39]. In the Spanish context, Tomás-Sábado [36] created and validated in Spanish The AIDS Attitude Scale (EASE) for nurses and nursing students. Several authors have used this tool in research, highlighting some limitations of the scale itself with the aim of improving it. Thus, Serrano-Gallardo et al. [40] and Leyva-Moral et al. [41] highlighted that the wording of some of the items generates ambiguity and comprehension problems, which could be affecting the internal consistency of the scale. On the other hand, Leyva-Moral et al. [41] and Álvarez-Serrano et al. [42] reported that, being a self-administered questionnaire, participants could answer according to what is considered a desirable image, i.e., accepted by the majority. Another consideration of Leyva-Moral et al. [41] is the date of creation of the questionnaire, which would imply a possible decontextualisation of the infection once it has become a chronic disease.

Empathy, in this case towards PLWHA, should be a basic competence in the nursing profession and should therefore be addressed as a priority during the undergraduate training of these professionals, as well as in later years [43]. On the other hand, the reduction or abolition of discriminatory practices and, therefore, the achievement of a positive environment for patients requires the improvement of attitudes of altruism, respect, solidarity, compassion and justice [24, 44]. Therefore, it is imperative to understand the attitudes of nursing students towards AIDS, with appropriate tools, in this new socio-cultural context of the disease. The objectives of this study were to update the original AIDS Attitudes Scale for Student Nurses (EASE) by adapting it to the sociocultural landscape and to analyse the reliability and structural validity of this new scale.

## Methods

### Design

This is a cross-sectional study that was carried out during the academic year 2020/2021 in the Nursing students of the Faculty of Health Sciences of the Ceuta campus of the University of Granada (Spain), for the validation study involving the adaptation of the some of the items of the original EASE scale [36].

### Sample, participants, and measures

The target population of the study consisted of those students enrolled in the Bachelor’s Degree in Nursing during the 2020/2021 academic year. They were summoned to an information session where the characteristics of the study were explained to them, and they were asked to participate voluntarily. In this way, a group of students was recruited to participate in the content validity and another group of students to take part in the pilot test-retest. The sampling was convenience sampling.

**Fig. 1:**
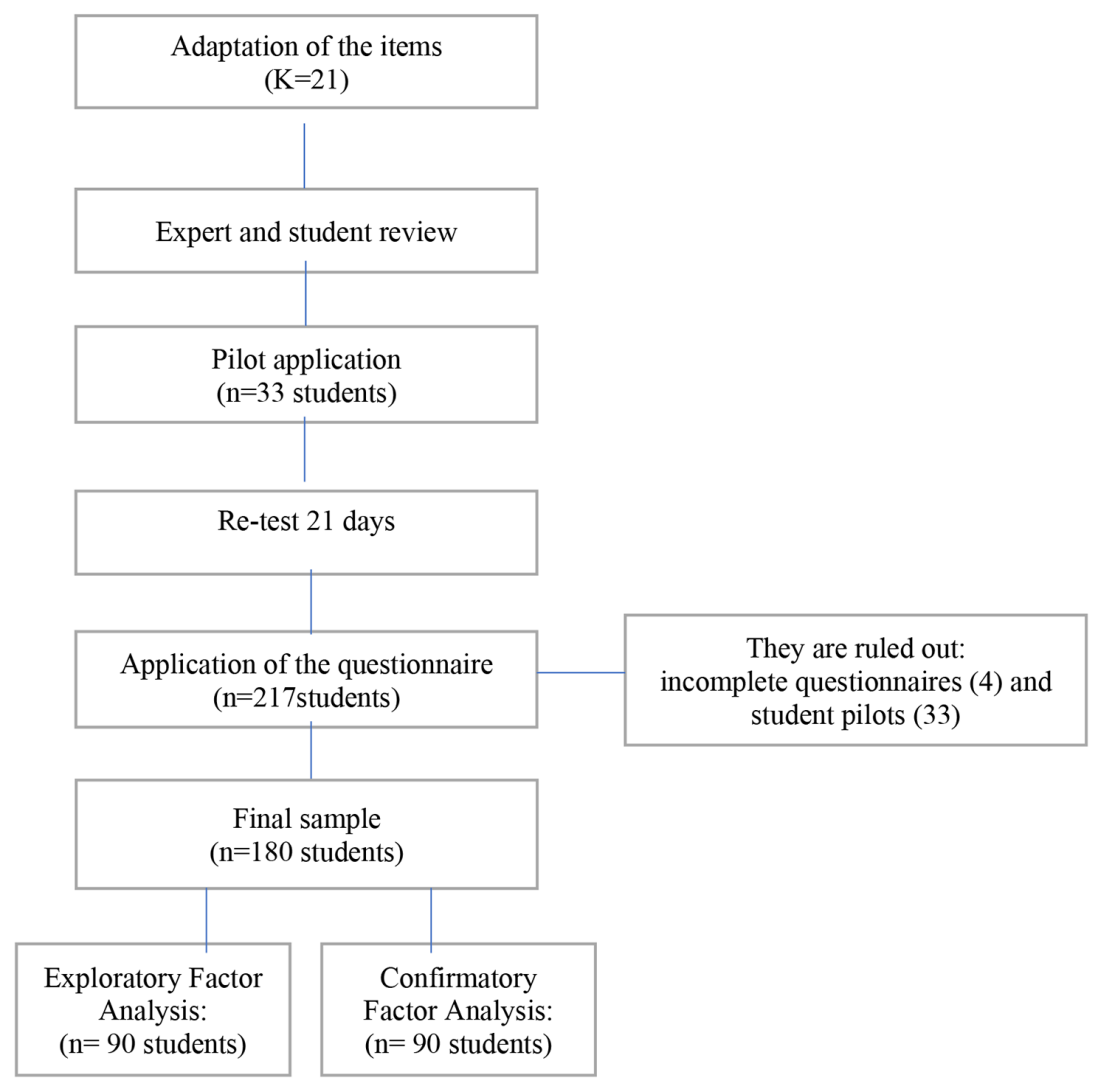
Diagram of the questionnaire design and implementation process

Once the pilot test was completed, the students of the Bachelor’s Degree in Nursing were contacted during class hours so that those who were interested could fill in the questionnaire. After excluding those who participated in the pilot test and the incomplete questionnaires (a total of four), the sample consisted of a total of 180 students, of which 50% of the questionnaires were used for the exploratory factor analysis (four participants per item included in the questionnaire), and the remaining 50% for the confirmatory factor analysis (Fig. 1). The Google Forms® tool was used for data collection.

### The instrument

In order to carry out this study, the original EASE scale proposed by Tomás-Sábado [36] was used as a reference. It is a scale that measures nursing students’ attitudes towards AIDS and consists of 21 items. The scale showed acceptable psychometric properties with a Cronbach’s alpha index of 0.779. At the time, the author of the scale emphasised that it should be a scale in a state of revision, given that the attitudes that are intended to be measured are related to highly changeable social and individual factors [36].

The adaptation to the new version of the scale was carried out by a group of experts in Health Sciences, university teaching and non-discriminatory language, who adapted it to achieve a more inclusive language adapted to the new socio-cultural context of this chronic disease. The content validity was carried out by a group of experts in the field and students of the Bachelor’s Degree in Nursing. The result was a provisional instrument that has been named the Attitudes towards People Living with HIV/AIDS Scale for Nursing Students (EAPVVS-E), also composed of 21 items. The questionnaire administered to participants contained, in addition to EAPVVS-E, socio-demographic variables (gender, age, marital status) and academic-cultural variables (course and religious beliefs).

### Ethical considerations

This study was approved by the Provincial Ethics Committee of Granada (Spain) with code 0568-N-22. All participants were provided with a written information sheet and signed informed consent. The right to refuse participation, to decline to answer questions posed or to withdraw at any stage of the process without any penalty or consequence was assured prior to eliciting participation. The author of the original scale was contacted to request permission for the adaptation and validation of the questionnaire. The ethical principles of the Declaration of Helsinki and the Oviedo Convention of Human Rights and Biomedicine were followed.

### Data analysis

Descriptive analyses and exploratory factor analysis (EFA) were performed with IBM® SPSS® Statistics version 25 for Macintosh®. The McDonald’s Omega coefficient was calculated by means of a self-developed tool using the Excel programme of the Microsoft® package. The confirmatory factor analysis (CFA) was carried out using IBM® SPSS® Amos version 24 for Windows®.

#### Validation of the instrument

To validate the EAPVVS-E scale, its psychometric properties were assessed using tests of content validity, internal consistency, test-retest stability, construct validity, convergent validity and discriminant validity.

In the process of adapting the EASE scale to the current sociocultural context, the expert group took into account the discrepancies found by other authors. The expert group was composed of nurses with professional experience in both clinical and teaching settings and experts in non-discriminatory inclusive language, who were familiar with the work of Tomás-Sábado [36]. These characteristics brought rigour and consistency to the sociocultural adaptation and validation process and helped to maintain a close link between the meaning of the items and the construct being explored. Content validity was assessed, in addition to the group of experts, by a group of ten students with sociodemographic characteristics similar to those of the final sample.

Stability or reliability was assessed by test-retesting a group of 33 students with similar socio-demographic characteristics to the final sample over a 21-day interval. This group of students was excluded from the factor analysis.

Construct validity was assessed by exploratory and confirmatory factor analysis in two stages. To identify the factors of the questionnaire, the data set was divided into two parts: n1 = 90 and n2 = 90 (50% and 50%, respectively). Exploratory factor analyses were conducted on the first part of the sample to identify factors, and confirmatory factor analyses were conducted on the second part of the data to confirm these factors. Exploratory factor analyses were conducted using the principal component analysis (PCA) method with varimax rotation. Each factor in the questionnaire was modelled as a variable. The number of factors was determined for *eigenvalues* greater than 1.

To estimate the reliability of the questionnaire, the internal consistency of each factor was measured by calculating the McDonald Omega coefficient (ω) [45]. This coefficient, unlike Cronbach’s alpha coefficient, works with factor loadings [46], which are the weighted sum of the standardised variables, a transformation that makes the calculations more stable [47] and reflects the true level of reliability. To be considered an acceptable value of reliability using the Omega coefficient, these must be between 0.70 and 0.90 [48], although in some circumstances values higher than 0.65 can be accepted [49].

The average variance extracted per factor (AVE) was calculated to estimate the convergent validity of the instrument. An AVE greater than 0.5 indicates that the measurement questions are better able to reflect the characteristics of each variable in the model [50]. To assess the discriminant validity between two factors, the shared variance (ϕ^2^ ) between them and the AVE per factor were taken into account, so that internal discriminant validity was considered to exist when the variance retained by each factor is greater than the shared variance between them (AVE > ϕ^2^ ) [50]. Finally, to determine the overall fit of the proposed model, a confirmatory factor analysis was performed with the maximum likelihood estimator on the covariance matrix. A parallel confirmatory factor analysis was performed with the structure marked by Tomás-Sábado [36] one-factor analysis was performed to compare results. The goodness-of-fit indices of this measurement model were analysed. Chi-square statistics and the CMIN/DF value (Discrepancy between chi-square and degrees of freedom) were used to assess the model fit [51, 52]. The comparative fit index (CFI) compares the model fit with that of an independent (zero) model, with a value above 0.90 indicating a good fit. The root mean square residual index (RMR) is based on the fitted residuals, it is recommended to be close to 0.5 with a value of less for a good fit [51]. For the root mean square error of approximation (RMSEA), a value of less than 0.08 indicates a reasonable fit. The adjusted goodness-of-fit index (AGFI), the normalised fit index (NFI) and the non-normalised fit index NNFI or TLI were measured for which values > 0.8 are acceptable and values > 0.9 are desirable [51].

## Results

### Description of the study sample

Table 1 presents the characteristics of the participants in the validation of the questionnaire. The pilot sample consisted of 33 s-year students with an average age of 21.76 years, 78.8% of whom were female. On the other hand, of the 180 participants selected for the factor analysis, 29.3% were in the first year, 58.3% were in the second year, 10.6% in the third year and 7.2% in the fourth year. 82.2% of the participants were female. The mean age of the participants was 22.22 years.

**Table 1:** Socio-demographic variables of the study population

	Pilot sample (n = 33)	Sample factor analysis (n = 180)
	**Mean (SD)**	**Mean (SD)**
Age (years)	21.76 (4.637)	22.22 (5.871)
	**n (%)**	**n (%)**
Sex		
Man	7 (21.2)	32 (17.8)
Woman	26 (78.8)	148 (82.2)
Academic year		
First	0	43 (29.3)
Second	33 (100)	105 (58.3)
Third	0	19 (10.6)
Fourth	0	13 (7.2)

Abbreviation: SD: Standard deviation.

### Content validity and reliability

The team of experts confirmed that all items proposed after adaptation of the questionnaire were clearly worded, relevant and consistent with the construct being measured, in this case, nursing students’ attitudes towards PLWHA. On the other hand, the stability of the questionnaire was assessed by test-retesting a group of 33 students with similar sociodemographic characteristics to those of the final sample over a 21-day interval, which resulted in a Cohen’s Kappa coefficient of 0.51.

### Construct validity, convergent validity and discriminant validity: exploratory factor analysis (EFA)

The adequacy of the factor analysis was confirmed by a Kaiser-Meyer-Olkin index of sampling adequacy which yielded a result of 0.83. Bartlett’s test of sphericity was significant (1086.986, df = 190, sig.= 0.001).

A first exploratory factor analysis showed the existence of 5 factors, however, items 3, 8 and 14 were eliminated as they had communalities lower than 0.4, while items 6, 7, 10 and 21 were eliminated as they had loadings lower than 0.5. In the final solution, *eigenvalues* greater than 1 showed the existence of four factors. This solution converged in five iterations and explains 60.99% of the variance. The items present factor loadings greater than 0.50 within their factor and communalities greater than 0.50 (Table 2). Factor loadings for all items were above the threshold of 0.40, furthermore, the AVE was above 0.5, indicating high convergent validity.

**Table 2:** Exploratory Factor Analysis (EFA) results

	Communalities	Factor 1	Factor 2	Factor 3	Factor 4
Item 1	0.612			0.746	
Item 2	0.629			0.784	
Item 4	0.512			0.595	
Item 5	0.627		0.772		
Item 9	0.527	0.649			
Item 11	0.654		0.792		
Item 12	0.665				0.714
Item 13	0.733				0.853
Item 15	0.515		0.645		
Item 16	0.602	0.761			
Item 17	0.765	0.810			
Item 18	0.565	0.705			
Item 19	0.621	0.769			
Item 20	0.510	0.673			
AVE		0.53	0.55	0.51	0.62

Abbreviation: AVE: Average variance extracted.

The four factors identified were qualitatively labelled by the judges and describe attitudes related to: professional practice, social integration, partner and family, and benevolent stigma.

Discriminant validity between two factors was determined when the values of variance retained by each factor are greater than the variance shared between them (AVE > ϕ2). The results obtained indicate validity between the 4 factors (Table 3).

**Table 3:** Internal and discriminant validity of the scale

		AVE_1_	AVE_2_	ϕ	ϕ^2^	
F.1	F.2	0.53	0.55	0.50	0.25	Yes
F.1	F.3	0.53	0.51	0.36	0.13	Yes
F.1	F.4	0.53	0.62	0.35	0.12	Yes
F.2	F.3	0.55	0.51	0.25	0.06	Yes
F.2	F.3	0.55	0.62	0.14	0.02	Yes
F.3	F.3	0.51	0.62	0.23	0.05	Yes

Abbreviations: F.1: Professional practice; F.2: Social integration; F.3: Couple and family; F.4: Benevolent stigma; AVE Average variance extracted; ϕ: Interfactorial correlation; ϕ^2^: Variance.

### Internal consistency

The internal consistency analysis is shown in Table 4. The McDonald’s Omega coefficient (ω) was 0.893 for the total scale. All factors scored above 0.75, which is considered an adequate result.

**Table 4:** Omega coefficient (ω) for the four factors of EAPVVS-E

Factor	Number of items	ω
F1. Professional practice	6	0.872
F2. Social integration	3	0.782
F3. Couple and family	3	0.754
F4. Benevolent stigma	2	0.763
Total scale	14	0.893

### Confirmatory factor analysis (CFA)

**Fig. 2:**
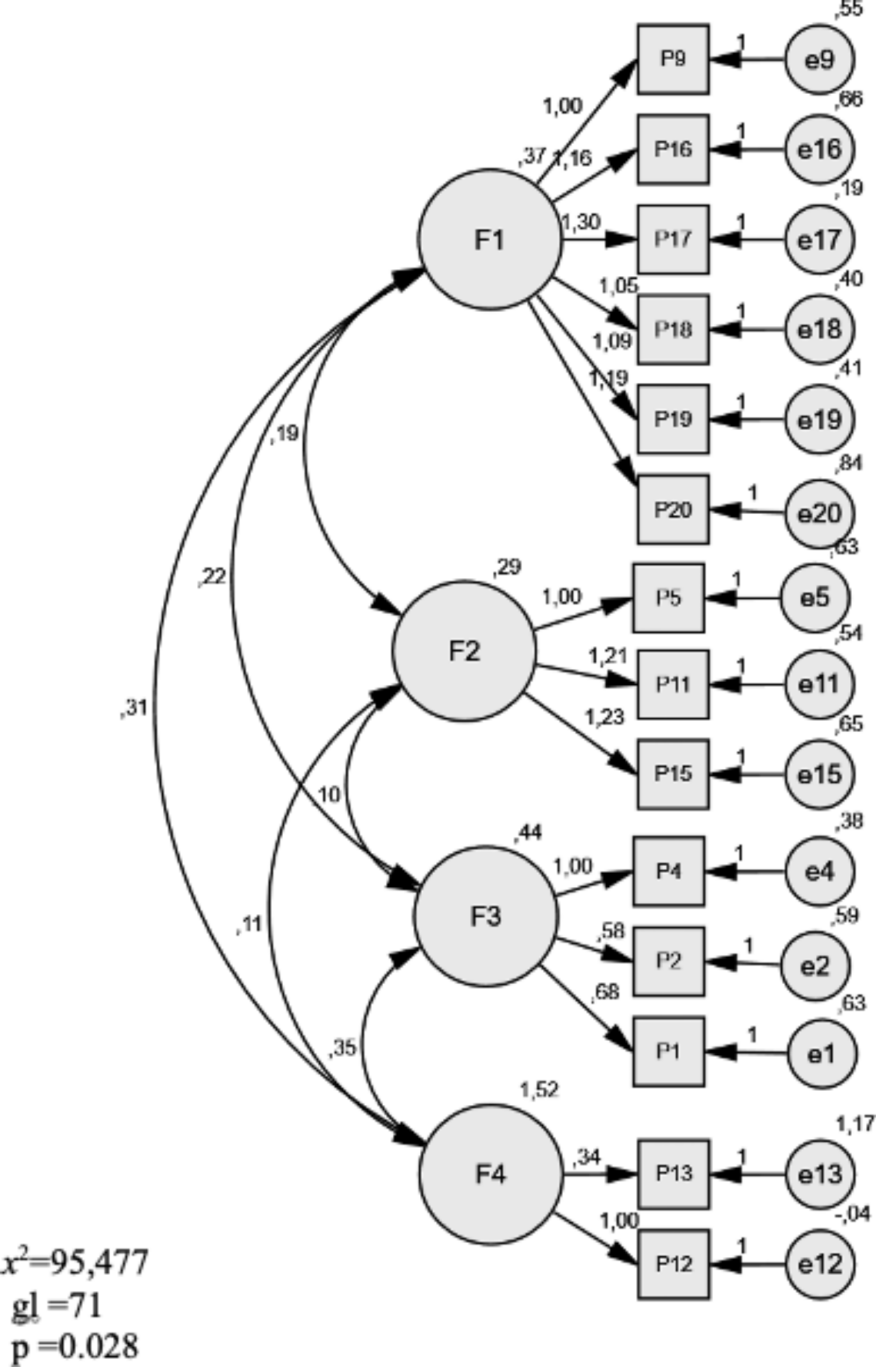
Confirmatory Factor Analysis of the four-factor model, with standardised weights and measurement errors for each of the items included in the EAPVVS-E scale

A confirmatory factor analysis was then carried out in order to test the exploratory factor structure of the 4-factor model. The results are compared with the one-factor model developed by Tomás-Sábado [36]. For the estimation of the goodness-of-fit parameters, the maximum likelihood method was used; the fit indexes [51, 52] are presented in Table 5. Figure 2 shows the model with the standardised scores. The chi-square values (χ^2^) are statistically significant in both models; however, the four-factor measurement model presents a better fit (Table 4), indicating that the items correctly reflected the latent constructs. The weighted fit index AGFI (0.897) is very close to the recommended cut-off value and considered satisfactory. The rest of the indices (RMSEA, CMIN/DF, GFI, CFI, TLI) also met the recommended criteria.

**Table 5:** Expected fit indices for a structural equation model and indices obtained for the confirmatory factor analysis

Adjustment index	Expected	1 Factor Model	4 Factor Model
χ^2^	> 0,05	0.001	0.028
CMIN/DF	< 5	**2.506**	**1.345**
GFI	0.90–1	0.792	**0.930**
AGFI	> 0.8 / 0.90–1	0.743	**0.897**
RMR	≈ 0.5	0.101	**0.052**
RMSEA	< 0.05 / 0.08	0.092	**0.044**
IFC	> 0.8 / 0.90–1	0.733	**0.964**
NFI	> 0.8 / 0.90–1	0.629	**0.876**
TLI	> 0.8 / 0.90–1	0.702	**0.954**

Abbreviations: χ^2^: Chi-square; CMIN/DF: Discrepancy between chi-square and degrees of freedom; GFI: Goodness-of-fit index; AGFI: Weighted fit index; RMR: Root mean square residual index; RMSEA: Root mean square error of approximation; CFI: Comparative fit index; NFI: Normalised fit index; NNFI or TLI: Non-normalised fit index.

### Item analysis

The EAPVVS-E scale consists of 14 items distributed in four factors: (1) Professional practice: 6 items (9, 16, 17, 18, 19 and 20); (2) Social integration: 3 items (5, 11 and 15); (3) Partner and family: 3 items (1, 2 and 4); and (4) Benevolent stigma: 2 items (12 and 13). It is a self-administered questionnaire in which each item has five response options. Each factor is rated out of five. The directionality of the items is taken into account so that the higher the score, the more acceptable the respondent’s attitude is considered. The items of factors 1 (professional practice), 3 (partner and family) and 4 (benevolent stigma) are rated on a scale where 1 means strongly agree and 5 means strongly disagree; whereas the items of factor 2 (social integration) are rated inversely (1, strongly disagree; 5, strongly agree). Table 6 shows the results of the scores obtained in each of the factors of the questionnaire, so that the student body presents positive attitudes towards PLWHA, especially in factor 2 (social integration), while benevolent stigma is very present (3.60 points).

**Table 6:** Descriptive statistics of the EAPVVS-E

	Mean	SD
Factor 1. Professional practice	4.27	0.75
Factor 2. Social integration	4.45	0.77
Factor 3. Couple and family	4.39	0.66
Factor 4. Benevolent stigma	3.61	0.99

Abbreviation: SD: Standard deviation.

## Discussion

The aim of this study was to update the original Attitudes to AIDS in Nursing Students (EASE) scale by adapting it to the sociocultural landscape and to analyse the reliability and structural validity of this new scale. The original scale, although it demonstrated good psychometric properties in its validation process, is obsolete in terms of clinical vocabulary and inclusive language. Thus, the terms “AIDS virus”, “AIDS virus carriers”, or “AIDS patient”, among others, do not reflect the current reality in terms of the chronic nature of HIV infection [41] where, of 38.4 million people infected with the virus worldwide, only 1.7% died from AIDS-related illnesses in 2021, declining by 68% since 2010 [53]. In terms of inclusive language, it seems illogical for items to refer only to men, although the emergence of the first AIDS cases was reported only among men [2, 3].

At the time, the author of the original scale emphasised that it should be a scale in a state of revision, given that the validation of such a measuring instrument must incorporate observations derived from its practical use over time [34]. In this sense, both Serrano-Gallardo et al. [40] and Leyva-Moral et al. [41] and Álvarez-Serrano et al. [42] identified inconsistencies due to a certain degree of ambiguity in the wording of the items, which could have led to a lack of understanding of the questions by the participants that could be affecting the internal consistency of the scale. This new proposal was intended to solve these inconsistencies, however, according to the results of the validation, it confirms the opposite, that is, that there is stigma among the surveyed students in relation to the idea that specific hospitals should be created for people with AIDS and HIV and to the idea that HIV is the greatest plague of our time, specified in factor 4 of our scale. Therefore, these results suggest that the implementation of educational strategies focused on these attitudes should be a priority.

The original scale consisted of 21 items. The proposed new EAPVVS-E scale consists of 14 items. This new proposal arises from the need to update the measurement instrument to the current context while ensuring satisfactory psychometric properties. The new proposed EAPVVS-E offers several advantages over other instruments traditionally used to assess nursing students’ attitudes towards PLWHA [54–56]. First, the alignment of the proposed new model with the theoretical structure of the original EASE scale supports, to some extent, the structural validity of this new measurement approach. Secondly, the comparison of the fit indices of the single-factor model with the 4-factor model indicates a more satisfactory fit result with the EAPVVS-E model. This demonstrates the validity of the updated version of the instrument compared to the original one. Thirdly, all absolute fit indices met the recommended cut-off values, indicating the parsimony of the model. Fourth, the overall factor structure is consistent with expectations and the investigation of the parameters of the confirmatory factor analysis of the scales of these instruments supports the construct validity of the EAPVVS-E instrument. Fifth, the eigenvalues of the factors were greater than 1, and the internal reliability measured by McDonald’s Omega coefficients (ω) were at an adequate level, ranging from 0.75 to 0.87 for the EAPVVS-E scale. An acceptable range of Omega coefficient often cited is a value of 0.70 or higher [48]. Finally, based on the agreement regarding the appropriate range of acceptability, the EAPVVS-E scale provides an appropriate level of reliability.

The EAPVVS-E scale incorporates 4 factors not foreseen in the original scale: (a) Professional practice; (b) Social integration; (c) Partner and family; and (d) Benevolent stigma. This factorial structure allows, on the one hand, the clear delimitation of the different attitudinal elements according to the environment in which they occur. On the other hand, it will facilitate the establishment of monitoring indicators to specify the needs for educational intervention in this area, especially in factor 4. In this way, the EAPVVS-E scale can be used as a diagnostic tool that allows both students and teachers to identify areas of action on which to focus education on the prevention of stigma associated with PLWHA. According to the results obtained in this validation, these educational interventions should be carried out mainly on the benevolent stigma factor. Problem-solving educational interventions from a biomedical, cultural, and ethical perspective are needed to improve attitudes towards PLWHA.

### Limitations

This study has the following limitations that should be highlighted. Firstly, these results can only be taken as an exploratory approach, as the sample only included 180 nursing students. The sample consisted of students from different academic years; considering the academic year in the analyses, as well as whether or not students have had contact with external placements is necessary to identify whether it influences both professional practice-related competences and benevolent stigma. In addition, other educational categories such as nursing assistants or laboratory technicians, and professionals such as registered nurses, doctors and health technicians should be explored. Therefore, replication of these data in larger samples that include a greater diversity of educational and professional contexts is required. Secondly, both the original scale and this new proposal contain two-way items. Although, according to van Sonderen et al. [57], the inclusion of negatively worded items does not influence the response bias of the questionnaires, but rather the bias comes from the lack of attention of the respondents, it is preferable, for epidemiological and clinical studies, to develop short questionnaires (around 10 items) in the same direction. In this proposal, given the characteristics of the subject matter to be explored, it was not possible to ask all the questions in the same direction, but the number of items was reduced to 14 with respect to the original scale; in addition, a test-retest was carried out at an interval of 21 days.

On the other hand, Leyva-Moral et al. [41] and Álvarez-Serrano et al. [42] have highlighted that the use of self-administered questionnaires would be generating participants to answer according to what is considered a desirable image [58]. However, we believe that this method of data collection should be maintained, given the inherent characteristics of the questionnaire content, which measures attitudes towards people and clinical data with special protection of confidentiality. Furthermore, the results obtained for factor 4, benevolent stigma, do not seem to indicate that any response bias is occurring.

Despite these limitations, this proposed EAPVVS-E scale stands out for its sound psychometric properties. Its brevity, simplicity, as well as ease of correction and interpretation, make it a useful instrument for research use in educational and clinical nursing contexts.

## Conclusions

It is necessary to know the attitudes of nursing students towards people living with HIV/AIDS in the current socio-cultural context from the perspective of a stable chronic infection with antiretroviral treatment. Empathy, reflected in this specific case towards PLWHA, is a basic competence of the nursing profession and should therefore be developed throughout the Bachelor’s Degree in Nursing. The development of attitude assessment instruments will make it possible to implement specific educational actions that contribute to the elimination of the stigma of PLWHA and to improving the quality of nursing care. Specific knowledge of the four key factors of nursing students’ attitudes towards PLWHA proposed in this study may help to promote the design of more specific teaching-learning interventions in undergraduate programmes. In this line, the proposed EAPVVS-E scale has satisfactory psychometric properties, so it can be used both to assess the four attitudinal factors explored and to evaluate the impact of educational interventions. Therefore, this study opens the way for future research on the relationship between nursing education and students’ attitudes towards PLWHA.

## Electronic supplementary material

Below is the link to the electronic supplementary material.

Supplementary Material 1

## Data Availability

All data generated or analysed during this study are included in this published article and its supplementary information files.

## List of abbreviations

AGFI: Weighted fit index
AIDS: Acquired immunodeficiency syndrome
ART: Antiretroviral treatment
AVE: Average variance extracted per factor
CFI: Comparative fit index
CMIN/DF: Discrepancy between chi-square and degrees of freedom
DF: Degrees of freedom
EFA: Exploratory factor analysis
EAPVVS-E: Attitudes towards People Living with HIV/AIDS Scale for Nursing Students
EASE: AIDS Attitudes Scale for Student Nurses
GFI: Goodness-of-fit index
HIV: Human immunodeficiency virus
NFI: Normalised fit index
NNFI or TLI: Non-normalised fit index
PLWHA: People living with HIV/AIDS
RMR: Root mean square residual index
RMSEA: Root mean square error of approximation
χ^2^: Chi-square
