# Supplementary material for: Validation of the attitudes towards people living with HIV/AIDS scale in nursing students
Source: BMC Nurs. 2023 Jul 27;22:245. doi: 10.1186/s12912-023-01414-6 (PMC10373256; doi:10.1186/s12912-023-01414-6)
Supplement: Supplementary file 1 — Supplementary Material 1 [file 12912_2023_1414_MOESM1_ESM.docx]

**Supplementary material.** Attitudes Towards People Living with HIV/AIDS Scale for Nursing Students (EAPVVS-E)

| **Item 1** | HIV/AIDS does not affect heterosexual couples. |
| --- | --- |
| **Item 2** | Fetuses infected with the HIV virus should be aborted. |
| **Item 4** | HIV-positive women should not be allowed to become pregnant. |
| **Item 5** | HIV/AIDS is everyone's problem. |
| **Item 9** | HIV-infected persons should be isolated from other patients. |
| **Item 11** | Being a carrier of HIV should not be an obstacle to access to education and employment. |
| **Item 12** | Specific hospitals should be created for people with AIDS and HIV. |
| **Item 13** | HIV is the greatest plague of our time. |
| **Item 15** | Being a carrier of HIV should not be an impediment to adopting a child. |
| **Item 16** | In the hospital, a person carrying the HIV virus should not share a room with an uninfected person. |
| **Item 17** | I would not find it pleasant to work with a colleague who is HIV positive. |
| **Item 18** | Children carrying the HIV virus should attend special classes. |
| **Item 19** | As a precautionary measure, we should avoid contact with people with AIDS and HIV. |
| **Item 20** | Whenever we touch a person with AIDS, we should always wear gloves. |
